# The impact of avocado intake on anthropometric measures among Hispanic/Latino children and adolescents: A cluster randomized controlled trial

**DOI:** 10.1016/j.clnesp.2023.04.020

**Published:** 2023-05-03

**Authors:** Hannah VanEvery, Lorena S. Pacheco, Elizabeth Sun, Matthew A. Allison, Xiang Gao

**Affiliations:** a Department of Nutrition, Harvard T.H. Chan School of Public Health, Boston, MA, USA; b Clinical and Translational Epidemiology Unit, Massachusetts General Hospital, Boston, MA, USA; c Department of Family Medicine, University of California San Diego, La Jolla, CA, USA; d Department of Family Medicine, University of California San Diego School of Medicine, La Jolla, CA, USA; e Department of Nutrition and Food Hygiene, School of Public Health, Institute of Nutrition, Fudan University, Shanghai, China

**Keywords:** Nutrition, Childhood obesity, Avocado, Adiposity

## Abstract

**Background/Objective:**

While the association between avocado consumption and low metabolic risk has been shown in some studies conducted in adults, little is known about the potential effects of avocado consumption on health outcomes in children and adolescents. Thus, we investigated the impact of two levels of avocado allotment, plus a standard nutrition education, on measures of adiposity in children and adolescents (<18 years old).

**Methods:**

Children (aged 5–12, n = 58) and adolescents (aged 13–17, n = 32) in seventy-two families that self-identified as Hispanic, with at least 3 members over the age of 5 that resided in the same home, were free of severe chronic disease, and not on specific diets, were randomized to one of two levels of avocado allotment plus bi-weekly nutrition education sessions. Low allotment families received 3 avocados per week, while high allotment families received 14 avocados per week for 6 months. We performed an intention-to-treat analysis, using unpaired, 2-sided t-tests to test the mean changes in anthropometric measures of adiposity (body mass index (BMI), waist circumference, hip circumference, and weight) between children and adolescents from high and low allotment families after the 6-month intervention.

**Results:**

At six months, there were no significant differences in body mass index, waist circumference, hip circumference, or waist circumference to weight ratio by avocado allotment group. In children, there was a significant difference in weight (difference in means: 1.10, 95% CI: 0.09, 2.10, p-value = 0.03) and waist circumference to height ratio (difference in means: 0.27, 95% CI: 0.12, 0.41, p-value <0.01) between the avocado allotment groups at six months, but these did not remain significant after sensitivity analyses including perprotocol analyses. In adolescents only, there was a significant reduction in waist to hip circumference ratio in the high allotment group compared to the low allotment group after 6 months (difference in means: −0.05, 95% CI: −0.08, 0.00, p-value = 0.04) that persisted after multiple sensitivity analyses.

**Conclusions:**

Different levels of avocado availability among children and adolescents does not appear to result in significant changes in anthropometric measures. Further study is needed to determine whether avocado consumption promotes metabolic health in this age group.

## 1. Introduction

Excess adiposity in childhood and adolescence has been linked to unfavorable health outcomes, both during this period (e.g. metabolic syndrome [1], and later in adulthood (e.g. heart failure) [2]. Understanding the impact of dietary components on measures of adiposity and body composition in childhood and adolescence is an important first step towards developing strategies to prevent chronic diseases throughout the lifespan. This is especially relevant for Hispanic/Latino populations, as the prevalence of obesity is highest among Hispanic children and adolescents, compared to non-Hispanic white, non-Hispanic black, and non-Hispanic Asian children and adolescents in the United States [3].

The potential beneficial effects of avocado consumption in adult populations have been explored in both observational and interventional studies [4–7], but the impact of avocado consumption in children and adolescents remains largely unexplored. One publication described the differences between avocado consumers and non-consumers in American adolescents (12–18 years old) [8], and showed that while avocado consumers had significantly higher diet quality scores than non-avocado consumers, there were no significant differences in body mass index (BMI) z-score, weight to height ratio, or body fat percent between these two groups [8].

In adults, avocado consumption significantly reduced low-density lipoprotein cholesterol concentrations (LDL-c) in one clinical trial [5], while a separate trial found that a hypocaloric diet including an avocado a day resulted in weight loss that was not significantly different than a hypocaloric diet alone [6]. Observational studies have found that consumers of avocados had lower body weight, BMI, and waist circumference (WC) than their nonavocado consuming counterparts [7]. Evidence on the impact of avocado consumption on metabolic health outcomes in adults is emerging. It is possible that the high fiber and monounsaturated fat content of avocados leads to increased satiety, that the consumption of avocados impacts the gut microbiome, or that there are metabolic benefits to consuming the phytochemicals found in avocados. However, further exploration is needed to understand whether avocado consumption in early life promotes positive health outcomes.

In this context, we conducted a family-based randomized controlled trial to investigate the effects of avocado consumption on measures of adiposity among Hispanic children and adolescents. We hypothesized that children and adolescents in the high avocado allotment group would experience an improvement in measures of adiposity after the six-month intervention, compared to those assigned to the low avocado allotment.

## 2. Methods

### 2.1. Study design and population

The present study included participants from the Effects of Avocado Intake on the Nutritional Status of Families trial [9], which was a cluster, family-based, randomized controlled trial that took place in San Diego County, California. The details of the study design are published elsewhere [9]. In brief, families were randomized to either a low avocado (3/week) or high avocado (14/week) allotment for 6 months, and both groups were provided nutrition education. Clinic visits occurred at months zero, three, and six.

To be included in this trial, families had to meet the following criteria: be composed of 3–8 members over the age of 5 residing in the same home, be willing to participate, and self-identify as Hispanic/Latino. Families were not included if they contained a member with a clinical condition requiring a specific diet, any member had an avocado or latex allergy, any adult member consumed >1 avocado/day or any child member consumed >½ an avocado/day, any member was unwilling to consume avocados, any member was pregnant or planning to become pregnant, or any member was planning to move during the study period.

The study protocol and all study materials were approved by the Institutional Review Boards of the University of California San Diego and San Diego State University. This clinical trial was registered under clinicaltrials.gov study identifier NCT02903433. Families with members younger than five years old were included, but these members were not counted as family members and were not expected to participate in the intervention.

### 2.2. Setting, recruitment, consent, and randomization

From April 2017 to June 2018, participants were recruited in San Diego County [9].

After recruitment, potential families began a 14-day run-in period that assessed the family’s adherence to study procedures. Families were randomized to an intervention group if during this period they completed a home visit, all questionnaires, had their blood drawn (in adults), demonstrated a commitment and willingness to participate in the study, and scheduled a baseline clinic visit. The “head of the household” was identified at the baseline clinic visit and was defined as the family member that primarily shopped for groceries and prepared family meals.

Randomization of the family was performed using a blocked randomization sequence generated using SAS (SAS Institute, Inc., Cary, NC, USA). While all staff members and study researchers were blinded to the randomization outcome, community health workers and participants were unblinded due to the need to assist in the distribution of avocados and intervention delivery respectively [9].

### 2.3. Intervention

Families in both avocado allotment groups received identical nutrition education throughout the 6-month period that provided families with tools to improve diet quality and meet nutritional goals. This education was delivered by registered dietician-trained community health workers in the homes of the participants and consisted of 12 bi-weekly culturally and language appropriate nutrition education materials derived directly from resources provided by the United States Department of Agriculture MyPlate/MiPlato (http://www.choosemyplate.gov/; accessed on 11 March 2016). Details on the preparation and delivery of nutrition education have been previously published [9].

The families in the low avocado allotment group received 3 avocados/week, which was based on the average reported intake of avocados in the target population from survey data of 101 participants collected prior to initiation of the trial [9]. Providing avocados, as opposed to providing no intervention, was chosen to standardize the avocado consumption of the “control” group in this study, while not increasing or decreasing typical intake. Families in the high avocado allotment group received 14 avocados/week. This number of avocados was chosen to allow for a robust increase in avocado consumption [9]. Families in both groups were encouraged to not purchase additional avocados.

Community health workers delivered avocados to each participating family on a weekly basis throughout the 6-month intervention and provided an avocado care guide that described how to ensure the fruit gradually matured throughout the week until the next delivery. Participants were compensated ($100 to each family) at 3 and 6 months.

### 2.4. Measurements

At baseline, 3 and 6 months, participants completed clinical visits. These visits included a dietary assessment-a self-administered, web-based, VioScreen (VioCare, Inc., Princeton, NJ, USA) food frequency questionnaire (FFQ) [10], a survey packet collecting socio-demographic characteristics, lifestyle factors and behaviors, and avocado consumption behavior, measurements of blood pressure and anthropometrics, and the measurement of physical activity via the global physical activity questionnaire [9]. This clinic visit also included a scheduling of a blood draw at a local Laboratory Corporation of America Holdings (LabCorp) site (for adults only).

Weight was measured using a balance beam scale, height was measured using a stadiometer, and waist and hip circumference (HC) were measured using a semi-flexible tape measure, 2 cm above the iliac crest for waist and at the level of the widest circumference over the greater trochanters for hip [11]. Blood pressure was measured using an automated Omron device three times at least 1 min apart after the participant had been sitting for at least 5 min.

The Avocado Daily Diary was developed for this study, to specifically capture avocado daily consumption of the family (not individually) and determine intervention adherence. This diary was completed by the head of household and returned to the community health worker during the bi-weekly home visits or weekly avocado deliveries. A copy of the Avocado Daily Diary has been published [9].

Children and adolescents only completed the physical activity survey, dietary assessment, and measurements of blood pressure and anthropometrics. The head of household completed all previously mentioned assessments and other adults in the household completed all assessments except for the participant survey packet [9].

### 2.5. Outcomes

The primary outcomes of the original study were total energy intake and nutritional status assessed at the family level, which has been published elsewhere [9]. The primary outcomes for the current study included child and adolescent anthropometric measures (BMI, WC, HC) and calculated measures of body composition based on these measures (WC to height ratio, WC to HC ratio, WC to weight ratio). Blood pressure in children and adolescents was reported with the primary outcomes of this trial [9].

### 2.6. Intervention adherence

Family intervention adherence was determined using the Avocado Daily Diary designed to collect data on daily intake by the family. Each child and adolescent’s weekly avocado consumption was calculated using the FFQ and used as a continuous adherence value in the statistical models.

### 2.7. Statistical analyses

The sample size and power in this trial were based on the primary outcome of total intake of energy in kcal/family/day. A sample size of 60 families provided 80% power to detect a 375-kcal difference (equivalent to 1.5 medium avocados/family/day) at an alpha of 0.05 and a standard deviation (SD) of up to 500 kcal/family/day. The initial trial aimed to randomize 70 total families to allow for up to 15% attrition and a final evaluable sample of 60 families.

Descriptive statistics were utilized to outline the study population by avocado allotment group. The continuous variables were reported as mean and standard deviation (SD) and categorical variables were reported as frequencies and percentages. For continuous variables, normality was evaluated. An intention-to-treat approach with no covariate adjustment was used for the primary analyses. Missing data was imputed by carrying forward the last observation for that variable.

Two-sided t-tests were used to assess mean differences in anthropometric variables between avocado allotment groups. Then, changes from baseline to month 3 and from baseline to month 6 for the anthropometric variables were determined, and mean differences between avocado allotment groups were assessed with 2-sided t-tests. Mean difference and standard deviation (SD or 95% confidence intervals (CI) are presented, when appropriate. As baseline BMI was significantly different between avocado allotment groups in children, the above primary analyses were repeated with the addition of BMI as a covariate.

As a sensitivity analysis, we performed the original intention-to-treat analysis using a clustered approach, clustering on family. Then, we adjusted the primary intention-to-treat analysis for adherence and baseline total calorie intake. We further repeated the primary analysis, this time performing a per-protocol analysis. We performed a per-protocol analysis adjusting for baseline BMI and another analysis that adjusted for baseline calorie intake and adherence in those participants that completed the intervention at 6 months.

All analyses were 2-tailed and P values of less than 0.05 were considered statistically significant. These analyses were conducted with R version 4.1.2 (R Foundation for Statistical Computing, Vienna, Austria).

## 3. Results

**Fig. 1.**
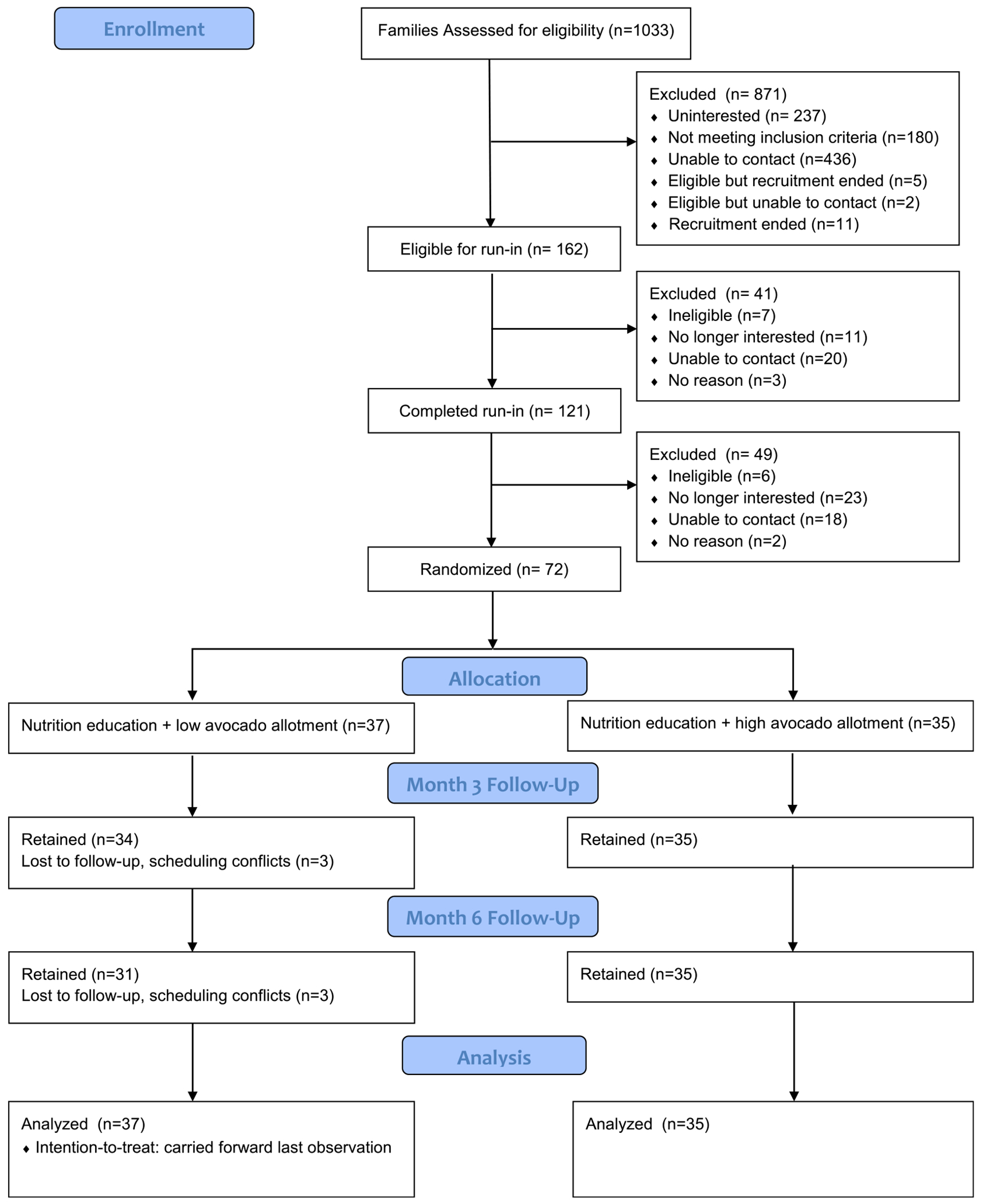
Consolidated standards of reporting trials (CONSORT) flow diagram of this secondary analysis of the Effects of Avocado Intake on the Nutritional Status of Families Trial.

This trial enrolled a total of 37 families in the low avocado allotment group and 35 families in the high avocado allotment group. Among the 58 children randomized (mean age 9.3 (SD 2.1); range 5–12 years), 30 children were assigned to the low avocado allotment group and 28 children were assigned to the high avocado allotment group (Table 1, Fig. 1). Among the 32 adolescents randomized (mean age 15.8 (SD 1.2); range 13–17 years), 14 adolescents were assigned to the low avocado group and 18 adolescents were assigned to the high avocado group (Table 1).

In children, the change in BMI, WC, HC, WC:HC, or WC:weight was not significantly different between the high and low avocado allotment groups at 6 months (Table 2). There was a significant difference in weight between the avocado allotment groups in children at six months. Both groups experienced a non-significant increase in weight (high allotment group: 3.06 kg, 95% CI: −6.78, 12.89 kg; low allotment group: 1.96 kg, 95% CI: −5.17, 9.20 kg), but there was a significant between-group difference (1.10 kg, 95% CI: 0.09, 2.10 kg, p-value = 0.03) (Table 2). There was also a significant difference in WC:height in children at six months (Table 2). Both groups experienced a significant increase in WC:height (high allotment group: 0.83, 95% CI: 0.74, 0.92; low allotment group: 0.56, 95% CI: 0.42, 0.69), and there was a significant between-group difference (0.27, 95% CI: 0.12, 0.41, p-value < 0.01) (Table 2). These results remained significant after a cluster-based analysis. For weight, the between-group difference was (1.30 kg, 95% CI: 0.19, 2.41 kg, p-value = 0.02). For WC:height, the between-group difference was 0.28 (95% CI: 0.10, 0.46, p-value <0.01). These results remained consistent after adjustment for baseline BMI (Table 3) and for baseline calorie intake and adherence (Table 4). However, these results did not remain significant in per-protocol analyses (Supplemental Tables S1–S3).

Among adolescents, the change in BMI, WC, weight, HC, WC:height, or WC:weight was not significantly different between the high and low avocado allotment groups at 6 months (Table 2). However, there was a significant difference in the mean change in WC:HC between the high avocado allotment and the low avocado allotment groups. The high avocado group experienced a non-significant decrease in WC:HC (−0.02, 95% CI: −0.06, 0.03) and the low avocado group experienced a non-significant increase in WC:HC (0.03, 95% CI: −0.03, 0.08) after this 6 month intervention. The between-group mean difference was significant (−0.05, 95% CI: −0.08, 0.00, p-value: 0.04) (Table 2). These results remained significant after a cluster-based analysis (−0.05, 95% CI: −0.10, 0.00, p-value: 0.04). The results were further consistent after adjustment for baseline BMI (Table 3) and baseline calorie intake and adherence (Table 4) and remained consistent in per-protocol analyses (Supplemental Tables S1–S3).

## 4. Discussion

In this cluster, family-based, randomized controlled trial among Hispanic/Latino families in San Diego, we found that a high allotment of 14 avocados per week, per family, was associated with a significant difference in the mean change in weight and WC:height between the high avocado allotment and the low avocado allotment groups in children that did not persist in secondary per-protocol analyses. In contrast, among adolescents, there was a significant difference in the mean change in WC:HC between the high avocado allotment and the low avocado allotment groups that was consistent in all analyses. Avocado allotment was not significantly associated with any other anthropometric measures.

There is a paucity of studies on the effects on avocado intake in children and adolescents, but our results are consistent with the one previously cross-sectional study [8]. In this study, Segovia-Siapco et al. collected dietary data via FFQ from 534 students (ages 12–18) at Adventist high schools in California and Michigan. These students also had anthropometric measures collected at school. The study authors used the dietary intake data to determine whether students were avocado consumers or non-consumers, and calculated the Diet Quality Index [12] for each student. The anthropometric data was used to calculate BMI-z score, waist to height ratio, fat mass, fat-free mass, and body fat percent. In these adolescents, avocado consumption was associated with a higher Diet Quality Index, but was not significantly associated with any of the measured or calculated anthropometric variables [8]. This is in agreement with observational studies of the consumption of other foods that, similar to avocados, are high in monounsaturated fatty acids [13,14]. Among Spanish children, exclusive olive oil consumption in salads and cooked foods, as opposed to consumption of other oils or a mixture of oils, was associated with a lower risk of increasing BMI z-score after 1 year [13]. Similarly, in a large observational study including children and adolescents from 18 countries, consuming 3 or more servings/week of nuts was associated with a lower BMI compared to those that consumed none or 1–2 servings/week [14].

In the current study, and while the majority of the measures explored were not associated with family avocado allotment, among adolescents, there was a consistent significant difference in the mean change in WC:HC between the high avocado allotment and the low avocado allotment groups. Notably, elevated WC:HC, or waist-to-hip ratio, has previously been associated with the metabolic risk in both adult [15] and child/adolescent [16] populations. As such, finding strategies to reduce WC:HC through diet are both important and timely.

Unlike adults, healthy children and adolescents are expected to gain weight, and to some extent height, over time. This was observed in the current study, in which weight and WC increased in both the high and low avocado allotment groups over the six-month intervention. In this study, there was a significant difference in the mean change in weight and HC:height in children only, and this did not persist in secondary analyses. These results are difficult to interpret, and the variation in weight and WC:height change may be due to differences in growth rates or the fact that only avocados were added to the diet, and broader dietary change was not enforced. While it is possible that specific diet or physical activity could impact adiposity, there were no baseline differences in physical activity or dietary intake between avocado allotment groups [9].

As no other outcomes were significantly associated with avocado allotment, these results should be interpreted with caution. Indeed, the results of our analyses require replication in larger cohorts focused on children and adolescents to explore whether avocado consumption impacts adiposity, and whether this effect varies by sex, age, or other participant characteristics. In these analyses, children and adolescents were analyzed separately, as adolescence is generally accompanied by a distinct hormonal, behavioral, and growth phenotype when compared to childhood [17–19]. However, it is not yet clear that this age distinction is consequential in the relationship between avocado consumption and adiposity. This study is limited by the increase in Type −1 error due to the fact that the initial trial was recruited and randomized based on the family unit and was not targeted at addressing the potential differences between adolescents and children.

Further, in this study we chose to use measures of adiposity including waist and hip circumference, and BMI, as opposed to BMI-z scores. While BMI z-scores are traditionally used to assess childhood adiposity, many other measures, including waist-to-height ratio [20,21], waist-to-weight ratio [20,22], and waist-to-hip ratio [20], have been shown to be associated with both metabolic outcomes and fat mass (as measured via DXA) in pediatric populations. Moreover, given the relatively short duration of this dietary intervention, using age and sex specific z-scores for this short-term dietary intervention would introduce unnecessary layers of transformations that would cloud the interpretation of the results [23].

Our study has several strengths, including the measurement of health outcomes associated with avocado intake in young participants, a currently underexplored area of nutrition research. Further, this intervention was culturally appropriate, and used bilingual community health workers and home visits to increase intervention adherence. This trial also had limitations. Specifically, it focused on one specific racial/ethnic group, and the results may not be applicable to other populations. The avocados also were provided at the family level, and while survey data indicated that children’s and adolescent’s adherence was acceptable, a controlled feeding trial or other tightly controlled intervention may provide further clarity on the association between avocado intake and health outcomes in young people. It is also possible that the intervention was not long enough to capture an effect of avocado consumption on measures of adiposity.

## 5. Conclusions

In conclusion, the present study demonstrates that a differential allotment of avocados may not impact measures of adiposity in children and adolescents. These results require further investigation and replication in order to fully understand the impact of avocado consumption on weight, adiposity, and metabolic risk factors in early life.

## Supplementary Material

supplementary material

## Figures and Tables

**Table 1:** Demographic and anthropometric measures at baseline in children and adolescents.

	Low Avocado N = 30	High Avocado N = 28
Children		
Female, n (%)	12 (40.0)	17 (60.7)
Family size, mean (SD)	3 (0.5)	3 (0.5)
Age, mean (SD)	9.0 (2.1)	9.4 (2.2)
Body mass index (kg/m^2^), mean (SD)	18.6 (3.2)	20.7 (5.7)
Waist circumference (cm), mean (SD)	64.7 (11.1)	68.2 (15.5)
Weight (kg), mean (SD)	35.9 (14.5)	39.5 (17.2)
Hip circumference (cm), mean (SD)	74.3 (11.6)	78.7 (14.7)
Waist circumference: Height (cm/cm), mean (SD)	0.48 (0.1)	0.50 (0.1)
Waist: Hip circumference (cm/cm), mean (SD)	0.88 (0.1)	0.87 (0.1)
Waist circumference: Weight (cm/kg), mean (SD)	1.99 (0.5)	1.87 (0.5)

Adolescents	Low Avocado N = 14	High Avocado N = 18

Female, n (%)	9 (69.2)	13 (72.2)
Family size, mean (SD)	3 (0.5)	3 (0.5)
Age, mean (SD)	16.0 (1.0)	15.6 (1.4)
Body mass index (kg/m^2^), mean (SD)	23.2 (5.7)	25.1 (4.6)
Waist circumference (cm), mean (SD)	80.9 (18.7)	84.0 (10.4)
Weight (kg), mean (SD)	59.5 (13.7)	64.6 (14.1)
Hip circumference (cm), mean (SD)	94.2 (9.4)	97.7 (8.5)
Waist circumference: Height (cm/cm), mean (SD)	0.48 (0.1)	0.50 (0.1)
Waist: Hip circumference (cm/cm), mean (SD)	0.81 (0.1)	0.84 (0.1)
Waist circumference: Weight (cm/kg), mean (SD)	1.31 (0.2)	1.22 (0.1)

a P-value for difference between high and low avocado groups.

**Table 2:** Changes in anthropometric variables among the adolescents and children in the Effects of Avocado Intake on the Nutritional Status of Families Trial.

	Within-group differences		Within-group differences		Between-group difference^a^		p-value
	Low Avocado		High Avocado				
	Mean	95% CI	Mean	95% CI	Mean	95% CI	
**Children and Adolescents Body mass index (kg/m^2^)**							
Difference at 3 months	0.20	−1.89, 2.30	0.20	−2.15, 2.55	0.00	−0.35, 0.35	0.99
Difference at 6 months	0.26	−1.80, 2.31	0.51	−1.89, 2.92	0.25	−0.47, 0.99	0.49
**Waist circumference (cm)**							
Difference at 3 months	0.79	−4.81, 6.34	2.65	−3.60, 8.90	1.86	−0.60, 4.38	0.13
Difference at 6 months	2.57	−2.87, 8.00	2.65	−3.35, 8.65	0.08	−4.41, 4.58	0.97
**Weight (kg)**							
Difference at 3 months	0.77	−6.59, 8.14	0.73	−7.50, 8.96	−0.04	−0.77, 0.68	0.90
Difference at 6 months	1.57	−5.80, 8.74	2.87	−5.52, 11.25	1.30	−0.16, 2.94	0.08
**Hip circumference (cm)**							
Difference at 3 months	1.76	−4.09, 7.61	1.49	−4.61, 7.59	−0.27	−1.70, 1.15	0.71
Difference at 6 months	2.49	−3.26, 8.17	2.84	−3.89, 9.07	0.35	−4.15, 4.92	0.87
**Hip circumference (cm)**							
Difference at 3 months	0.54	0.43, 0.65	0.76	0.68, 0.84	0.22	0.09, 0.34	<0.01
Difference at 6 months	0.56	−0.45, 0.67	0.76	0.68, 0.83	0.20	0.07, 0.32	<0.01
**Waist: Hip circumference (cm/cm)**							
Difference at 3 months	−0.01	−0.04, 0.02	0.01	−0.02, 0.05	0.02	−0.01, 0.05	0.13
Difference at 6 months	0.00	−0.02, 0.03	0.00	−0.03, 0.04	0.00	−0.03, 0.03	0.87
**Waist circumference: Weight (cm/kg)**							
Difference at 3 months	−0.03	−0.23, 0.17	0.02	−0.17, 0.21	0.05	−0.01, 0.11	0.08
Difference at 6 months	−0.01	−0.22, 0.20	0.00	−0.22, 0.23	0.01	−0.11, 0.13	0.84

	Low Avocado		High Avocado		Between-group difference		p-value
	Mean	95% CI	Mean	95% CI	Mean	95% CI	

**Children only Body mass index (kg/m^2^)**							
Difference at 3 months	0.19	−1.90, 2.27	0.30	−2.87, 3.46	0.11	−0.35, 0.57	0.63
Difference at 6 months	0.42	−1.71, 2.55	0.23	−2.93, 3.38	−0.19	−0.99, 0.60	0.62
**Waist circumference (cm)**							
Difference at 3 months	0.77	−5.57, 7.12	3.37	−5.06, 11.80	2.60	−1.02, 6.21	0.15
Difference at 6 months	2.90	−3.37, 9.17	4.00	−4.24, 12.24	1.10	−6.24, 8.43	0.76
**Weight (kg)**							
Difference at 3 months	0.97	−6.23, 8.16	1.24	−8.49, 10.97	0.27	−0.60, 1.14	0.54
Difference at 6 months	1.96	−5.17, 9.20	3.06	−6.78, 12.89	1.10	0.09, 2.10	0.03
**Hip circumference (cm)**							
Difference at 3 months	2.11	−3.87, 8.10	2.02	−6.04, 10.07	−0.09	−1.74, 1.55	0.91
Difference at 6 months	3.74	−2.36, 9.84	2.67	−5.28, 10.61	−1.07	−8.60, 6,45	0.77
**Waist circumference: Height (cm/cm)**							
Difference at 3 months	0.54	0.41, 0.67	0.81	0.72, 0.91	0.27	0.13, 0.41	<0.01
Difference at 6 months	0.56	0.42, 0.69	0.83	0.74, 0.92	0.27	0.12, 0.41	<0.01
**Waist: Hip circumference (cm/cm)**							
Difference at 3 months	−0.02	−0.05, 0.02	0.02	−0.04, 0.07	0.04	−0.01, 0.08	0.17
Difference at 6 months	−0.01	−0.04, 0.03	0.02	−0.03, 0.06	0.03	−0.03, 0.07	0.35
**Waist circumference: Weight (cm/kg)**							
Difference at 3 months	−0.05	−0.27, 0.17	0.02	−0.23, 0.26	0.07	−0.02, 0.16	0.15
Difference at 6 months	−0.02	0.26, 0.22	0.03	−0.29, 0.34	0.05	−0.15, 0.25	0.63

	Low Avocado		High Avocado		Between-group difference		p-value
	Mean	95% CI	Mean	95% CI	Mean	95% CI	

**Adolescents only Body mass index (kg/m^2^)**							
Difference at 3 months	0.24	−4.03, 4.51	0.08	−3.24, 3.29	−0.16	−0.75, 0.42	0.57
Difference at 6 months	−0.08	−4.15, 3.99	0.91	−2.55, 4.36	0.99	−0.41, 2.38	0.16
**Waist circumference (cm)**							
Difference at 3 months	0.73	−8.73, 10.20	1.68	−6.45, 9.80	0.95	−2.49, 4.37	0.58
Difference at 6 months	1.87	−7.18, 10.92	0.83	−6.74, 8.39	−1.04	−4.84, 2.76	0.58
**Weight (kg)**							
Difference at 3 months	0.37	−9.80, 10.55	0.05	−9.04, 9.13	−0.32	−1.52, 0.86	0.58
Difference at 6 months	0.47	−9.26, 10.19	2.61	−6.86, 12.08	2.14	−1.40, 5.69	0.22
**Hip circumference (cm)**							
Difference at 3 months	1.03	−5.83, 7.90	0.78	−4.69, 6.24	−0.25	−2.85, 2.34	0.84
Difference at 6 months	−0.20	−6.84, 6.44	3.08	−2.87, 9.02	3.28	−0.18, 6.73	0.06
**Waist circumference: Height (cm/cm)**							
Difference at 3 months	0.55	0.33, 0.77	0.68	0.54, 0.83	0.13	−0.11, 0.38	0.27
Difference at 6 months	0.56	0.35, 0.77	0.67	0.53, 0.80	0.11	−0.12, 0.34	0.34
**Waist: Hip circumference (cm/cm)**							
Difference at 3 months	0.00	−0.05, 0.05	0.01	−0.04, 0.06	0.01	−0.03, 0.05	0.66
Difference at 6 months	0.03	−0.03, 0.08	−0.02	−0.06, 0.03	−0.05	−0.08, 0.00	0.04
**Waist circumference: Weight (cm/kg)**							
Difference at 3 months	0.01	−0.12, 0.13	0.02	−0.07, 0.11	0.01	−0.03, 0.07	0.51
Difference at 6 months	0.02	−0.10, 0.13	−0.03	−0.13, 0.08	−0.05	−0.12, 0.03	0.23

a The between-group difference is calculated by subtracting the value of the low avocado group from the high avocado group.

**Table 3:** Changes in anthropometric variables, adjusted for body mass index, among the adolescents and children in the Effects of Avocado Intake on the Nutritional Status of Families Trial.

	Within-group differences		Within-group differences		Between-group difference^a^		p-value
	Low Avocado		High Avocado				
	Mean	95% CI	Mean	95% CI	Mean	95% CI	
**Children and Adolescents Body mass index (kg/m^2^)**							
Difference at 3 months	0.20	0.01, 0.40	0.20	−0.09, 0.50	0.00	−0.35, 0.36	0.99
Difference at 6 months	0.26	0.00, 0.52	0.51	−0.16, 1.19	0.25	−0.48, 0.99	0.49
**Waist circumference (cm)**							
Difference at 3 months	0.76	−2.11, 3.64	2.65	−0.78, 6.08	1.89	−0.58, 4.36	0.13
Difference at 6 months	2.57	−0.56, 5.69	2.65	−1.78, 7.07	0.08	−4.31, 4.47	0.97
**Weight (kg)**							
Difference at 3 months	0.77	−3.05, 4.60	0.73	−3.15, 4.61	−0.04	−0.78, 0.69	0.90
Difference at 6 months	1.47	−2.32, 5.27	2.87	−1.25, 6.98	1.39	−0.17, 2.96	0.08
**Hip circumference (cm)**							
Difference at 3 months	1.76	−1.37, 4.89	1.49	−1.28, 4.26	−0.27	−1.70, 1.16	0.71
Difference at 6 months	2.46	−0.94, 5.85	2.84	−1.46, 7.14	0.38	−3.96, 4.73	0.86
**Waist circumference: Height (cm/cm)**							
Difference at 3 months	0.54	0.43, 0.65	0.76	0.68, 0.83	0.21	0.09, 0.33	<0.01
Difference at 6 months	0.56	0.45, 0.67	0.75	0.68, 0.83	0.19	0.07, 0.32	<0.01
**Waist: Hip circumference (cm/cm)**							
Difference at 3 months	−0.01	−0.04, 0.02	0.01	−0.02, 0.05	0.02	−0.01, 0.05	0.12
Difference at 6 months	0.00	−0.02, 0.03	0.00	−0.03, 0.04	0.00	−0.04, 0.03	0.87
**Waist circumference: Weight (cm/kg)**							
Difference at 3 months	−0.03	−0.18, 0.12	0.02	−0.11, 0.16	0.05	−0.01, 0.11	0.08
Difference at 6 months	−0.01	−0.16, 0.15	0.00	−0.16, 0.17	0.01	−0.11, 0.13	0.84

	Low Avocado		High Avocado		Between-group difference		p-value
	Mean	95% CI	Mean	95% CI	Mean	95% CI	

**Children only Body mass index (kg/m^2^)**														
Difference at 3 months	0.19	−0.07, 0.45	0.30	−0.08, 0.67	0.11	−0.34, 0.56	0.63
Difference at 6 months	0.42	0.10, 0.74	0.23	−0.50, 0.95	−0.21	−0.96, 0.56	0.61
**Waist circumference (cm)**							
Difference at 3 months	0.77	−1.90, 3.45	3.37	−2.05, 8.79	2.60	−0.65, 5.84	0.11
Difference at 6 months	2.90	−0.49, 6.29	4.00	−3.06, 11.06	1.10	−5.75, 7.94	0.75
**Weight (kg)**							
Difference at 3 months	0.97	−2.11, 4.05	1.24	−2.84, 5.31	0.27	−0.58, 1.12	0.53
Difference at 6 months	1.96	−1.12, 5.04	3.06	−1.04, 7.15	1.10	0.14, 2.04	0.02
**Hip circumference (cm)**							
Difference at 3 months	2.11	−0.69, 4.92	2.02	−0.71, 4.75	−0.09	−1.72, 1.53	0.91
Difference at 6 months	3.74	0.06, 7.42	2.67	−3.51, 8.84	−1.07	−7.57, 5.42	0.74
**Waist circumference: Height (cm/cm)**							
Difference at 3 months	0.54	0.41, 0.67	0.81	0.74, 0.88	0.27	0.13, 0.42	<0.01
Difference at 6 months	0.56	0.42, 0.69	0.82	0.74, 0.90	0.26	0.11, 0.41	<0.01
**Waist: Hip circumference (cm/cm)**							
Difference at 3 months	−0.02	−0.05, 0.02	0.02	−0.04, 0.07	0.04	−0.01, 0.07	0.13
Difference at 6 months	−0.01	−0.04, 0.03	0.02	−0.03, 0.06	0.03	−0.02, 0.07	0.33
**Waist circumference: Weight (cm/kg)**							
Difference at 3 months	−0.05	−0.20, 0.11	0.02	−0.14, 0.18	0.07	−0.02, 0.15	0.12
Difference at 6 months	−0.02	−0.19, 0.15	0.03	−0.19, 0.25	0.05	−0.14, 0.24	0.62

	Low Avocado		High Avocado		Between-group difference		p-value
	Mean	95% CI	Mean	95% CI	Mean	95% CI	

**Adolescents only Body mass index (kg/m^2^)**							
Difference at 3 months	0.24	−0.05, 0.53	0.08	−0.43, 0.58	−0.16	−0.80, 0.47	0.60
Difference at 6 months	−0.08	−0.48, 0.32	0.91	−0.40, 2.21	0.99	−0.58, 2.55	0.21
**Waist circumference (cm)**							
Difference at 3 months	0.73	−5.75, 7.22	1.68	−1.72, 5.07	0.95	−2.83, 4.72	0.61
Difference at 6 months	1.87	−4.44, 8.17	0.83	−2.94, 4.59	−1.04	−4.74, 2.66	0.57
**Weight (kg)**							
Difference at 3 months	0.37	−6.02, 6.76	0.05	−3.42, 3.51	−0.32	−1.64, 0.98	0.61
Difference at 6 months	0.47	−5.70, 6.63	2.61	−1.93, 7.15	2.14	−1.85, 6.14	0.28
**Hip circumference (cm)**							
Difference at 3 months	1.03	−2.87, 4.94	0.78	−1.65, 3.20	−0.25	−3.10, 2.58	0.85
Difference at 6 months	−0.20	−4.05, 3.65	3.08	0.45, 5.70	3.28	−0.37, 6.92	0.08
**Waist circumference: Height (cm/cm)**							
Difference at 3 months	0.55	0.34, 0.76	0.68	0.55, 0.82	0.13	−0.09, 0.35	0.22
Difference at 6 months	0.56	0.35, 0.76	0.67	0.54, 0.80	0.11	−0.10, 0.32	0.30
**Waist: Hip circumference (cm/cm)**							
Difference at 3 months	0.00	−0.04, 0.05	0.01	−0.03, 0.05	0.01	−0.03, 0.05	0.69
Difference at 6 months	0.03	−0.02, 0.07	−0.02	−0.06, 0.02	−0.05	−0.08, −0.01	0.02
**Waist circumference: Weight (cm/kg)**							
Difference at 3 months	0.01	−0.10, 0.11	0.02	−0.05, 0.10	0.01	−0.04, 0.07	0.53
Difference at 6 months	0.02	−0.08, 0.11	−0.03	−0.11, 0.06	−0.05	−0.12, 0.04	0.27

a The between-group difference is calculated by subtracting the value of the low avocado group from the high avocado group.

**Table 4:** Changes in anthropometric variables, adjusted for baseline calorie intake and adherence, among the adolescents and children in the Effects of Avocado Intake on the Nutritional Status of Families Trial.

	Within-group differences		Within-group differences		Between-group difference^a^		p-value
	Low Avocado		High Avocado				
	Mean	95% CI	Mean	95% CI	Mean	95% CI	
**Children and Adolescents Body mass index (kg/m^2^)**							
Difference at 3 months	0.21	−2.03, 2.45	0.21	−1.99, 2.40	0.00	−0.38, 0.36	0.97
Difference at 6 months	0.24	−1.92, 2.40	0.52	−1.90, 2.94	0.28	−0.48, 1.04	0.46
**Waist circumference (cm)**							
Difference at 3 months	0.70	−5.19, 6.58	2.73	−3.08, 8.53	2.03	−0.60, 4.66	0.13
Difference at 6 months	2.43	−3.20, 8.07	2.90	−3.13, 8.93	0.47	−4.15, 5.09	0.84
**Weight (kg)**							
Difference at 3 months	0.81	−7.04, 8.67	0.75	−6.68, 8.17	−0.06	−0.83, 0.69	0.86
Difference at 6 months	1.45	−6.14, 9.04	2.86	−5.60, 11.32	1.41	−0.20, 3.02	0.09
**Hip circumference (cm)**							
Difference at 3 months	1.55	−4.62, 7.71	1.54	−4.10, 7.19	−0.01	−1.43, 1.42	0.99
Difference at 6 months	2.05	−3.88, 7.98	3.03	−3.21, 9.27	0.98	−3.67, 5.65	0.67
**Waist circumference: Height (cm/cm)**							
Difference at 3 months	0.57	0.46, 0.67	0.76	0.67, 0.84	0.19	0.07, 0.31	<0.01
Difference at 6 months	0.58	0.48, 0.68	0.75	0.68, 0.83	0.17	0.05, 0.30	<0.01
**Waist: Hip circumference (cm/cm)**							
Difference at 3 months	−0.01	−0.04, 0.02	0.01	−0.02, 0.05	0.02	−0.01, 0.05	0.18
Difference at 6 months	0.01	−0.02, 0.04	0.00	−0.03, 0.04	−0.01	−0.04, 0.03	0.76
**Waist circumference: Weight (cm/kg)**							
Difference at 3 months	−0.03	−0.24, 0.18	0.02	−0.16, 0.20	0.05	−0.01, 0.11	0.08
Difference at 6 months	−0.01	−0.23, 0.21	0.01	−0.22, 0.24	0.02	−0.11, 0.15	0.77

	Low Avocado		High Avocado		Between-group difference		p-value
	Mean	95% CI	Mean	95% CI	Mean	95% CI	

**Children only Body mass index (kg/m^2^)**							
Difference at 3 months	0.20	−2.05, 2.45	0.31	−2.73, 3.34	0.11	−0.37, 0.59	0.65
Difference at 6 months	0.40	−1.81, 2.61	0.22	−2.59, 3.03	−0.18	−0.98, 0.62	0.65
**Waist circumference (cm)**							
Difference at 3 months	0.76	−5.96, 7.48	3.54	−4.43, 11.51	2.78	−0.80, 6.36	0.12
Difference at 6 months	2.72	−3.84, 9.29	4.50	−3.12, 12.12	1.78	−5.44, 9.00	0.62
**Weight (kg)**							
Difference at 3 months	1.03	−6.69, 8.76	1.28	−7.52, 10.08	0.25	−0.66, 1.16	0.58
Difference at 6 months	1.96	−5.53, 9.45	3.05	−5.65, 11.76	1.09	0.01, 2.18	0.05
**Hip circumference (cm)**							
Difference at 3 months	1.78	−4.58, 8.13	2.13	−5.23, 9.50	0.35	−1.18, 1.90	0.64
Difference at 6 months	3.21	−3.18, 9.59	3.00	−4.28, 10.28	−0.21	−7.63, 7.21	0.96
**Waist circumference: Height (cm/cm)**							
Difference at 3 months	0.58	0.46, 0.70	0.81	0.72, 0.90	0.23	0.10, 0.37	<0.01
Difference at 6 months	0.59	0.47, 0.71	0.82	0.73, 0.90	0.23	0.08, 0.38	<0.01
**Waist: Hip circumference (cm/cm)**							
Difference at 3 months	−0.01	−0.05, 0.03	0.02	−0.04, 0.07	0.03	−0.02, 0.07	0.21
Difference at 6 months	0.00	−0.04, 0.03	0.02	−0.03, 0.07	0.02	−0.03, 0.07	0.41
**Waist circumference: Weight (cm/kg)**							
Difference at 3 months	−0.05	−0.29, 0.19	0.02	−0.22, 0.26	0.07	−0.02, 0.16	0.13
Difference at 6 months	−0.02	−0.28, 0.23	0.04	−0.28, 0.35	0.06	−0.15, 0.27	0.56

	Low Avocado		High Avocado		Between-group difference		p-value
	Mean	95% CI	Mean	95% CI	Mean	95% CI	

**Adolescents only Body mass index (kg/m^2^)**							
Difference at 3 months	0.24	−3.25, 3.74	0.08	−3.03, 3.18	−0.16	−0.81, 0.47	0.60
Difference at 6 months	−0.08	−4.21, 4.05	0.91	−2.54, 4.35	0.99	−0.60, 2.57	0.21
**Waist circumference (cm)**							
Difference at 3 months	0.57	−8.27, 9.41	1.68	−5.83, 9.18	1.11	−2.59, 4.80	0.55
Difference at 6 months	1.87	−6.92, 10.65	0.83	−6.86, 8.51	−1.04	−5.07, 2.99	0.60
**Weight (kg)**							
Difference at 3 months	0.36	−8.68, 9.40	0.05	−7.75, 7.84	−0.31	−1.62, 0.99	0.63
Difference at 6 months	0.47	−9.26, 10.20	2.61	−6.80, 12.02	2.14	−1.88, 6.17	0.29
**Hip circumference (cm)**							
Difference at 3 months	1.07	−4.56, 6.70	0.78	−4.16, 5.71	−0.29	−3.30, 2.70	0.84
Difference at 6 months	−0.20	−6.79, 6.39	3.08	−2.83, 8.98	3.28	−0.43, 6.98	0.08
**Waist circumference: Height (cm/cm)**							
Difference at 3 months	0.55	0.32, 0.77	0.68	0.54, 0.83	0.13	−0.10, 0.38	0.24
Difference at 6 months	0.56	0.35, 0.76	0.67	0.53, 0.80	0.11	−0.12, 0.34	0.33
**Waist: Hip circumference (cm/cm)**							
Difference at 3 months	0.00	−0.05, 0.05	0.01	−0.04, 0.06	0.01	−0.03, 0.05	0.61
Difference at 6 months	0.03	−0.02, 0.07	−0.02	−0.06, 0.03	−0.05	−0.09, 0.00	0.04
**Waist circumference: Weight (cm/kg)**							
Difference at 3 months	0.00	−0.12, 0.12	0.02	−0.05, 0.10	0.02	−0.03, 0.07	0.45
Difference at 6 months	0.02	−0.10, 0.13	−0.03	−0.13, 0.08	−0.05	−0.12, 0.04	0.28

a The between-group difference is calculated by subtracting the value of the low avocado group from the high avocado group.

## Data Availability

Data described in the manuscript, code book, and analytic code will be made available upon request pending application and approval. Proposals should be directed to mallison@health.ucsd.edu.

## Appendix A. Supplementary data

Supplementary data to this article can be found online at https://doi.org/10.1016/j.clnesp.2023.04.020.
