# Supplementary material for: The impact of avocado intake on anthropometric measures among Hispanic/Latino children and adolescents: A cluster randomized controlled trial
Source: Clin Nutr ESPEN. Author manuscript; Available in PMC 2026 Aug 14. (PMC13475448; doi:10.1016/j.clnesp.2023.04.020)
Supplement: Supplementary file 1 [file NIHMS2198813-supplement-supplementary_material.docx]

**Supplemental Table S1**. Changes in anthropometric variables among the adolescents and children in the Effects of Avocado Intake on the Nutritional Status of Families Trial, in a per protocol analysis.

|  | **Within-group differences** | | **Within-group differences** | |  | |  |
| --- | --- | --- | --- | --- | --- | --- | --- |
|  | **Low Avocado** | | **High Avocado** | | **Between-group difference^a^** | | **p-value** |
|  | **Mean** | **95% CI** | **Mean** | **95% CI** | **Mean** | **95% CI** |  |
| **Children and Adolescents** |  |  |  |  |  |  |  |
| **Body mass index (kg/m^2^)** |  |  |  |  |  |  |  |
| Difference at 3 months | 0.27 | -1.93, 2.47 | 0.21 | -2.19, 2.61 | -0.06 | -0.46, 0.33 | 0.76 |
| Difference at 6 months | 0.31 | -1.91, 2.52 | 0.52 | -1.94, 2.98 | 0.21 | -0.57, 0.99 | 0.60 |
|  |  |  |  |  |  |  |  |
| **Waist circumference (cm)** |  |  |  |  |  |  |  |
| Difference at 3 months | 0.97 | -5.54, 7.50 | 2.83 | -3.78, 9.44 | 1.86 | -0.90, 4.62 | 0.18 |
| Difference at 6 months | 3.44 | -3.11, 9.99 | 2.58 | -3.77, 8.93 | -0.86 | -5.96, 4.24 | 0.74 |
|  |  |  |  |  |  |  |  |
| **Weight (kg)** |  |  |  |  |  |  |  |
| Difference at 3 months | 1.01 | -7.62, 9.65 | 0.74 | -7.64, 9.14 | -0.27 | -1.08, 0.54 | 0.51 |
| Difference at 6 months | 1.92 | -6.90, 10.73 | 2.86 | -5.69, 11.41 | 0.95 | -0.71, 2.60 | 0.26 |
|  |  |  |  |  |  |  |  |
| **Hip circumference (cm)** |  |  |  |  |  |  |  |
| Difference at 3 months | 2.25 | -4.33, 8.83 | 1.59 | -4.92, 8.10 | -0.66 | -2.24, 0.92 | 0.41 |
| Difference at 6 months | 3.13 | -3.48, 9.75 | 2.83 | -3.81, 9.47 | -0.30 | -5.41, 4.80 | 0.91 |
|  |  |  |  |  |  |  |  |
| **Waist circumference: Height (cm/cm)** |  |  |  |  |  |  |  |
| Difference at 3 months | 0.74 | 0.67, 0.80 | 0.81 | 0.74, 0.88 | 0.07 | 0.01, 0.14 | 0.02 |
| Difference at 6 months | 0.76 | 0.69, 0.82 | 0.80 | 0.74, 0.87 | 0.05 | -0.02, 0.11 | 0.17 |
|  |  |  |  |  |  |  |  |
| **Waist: Hip circumference (cm/cm)** |  |  |  |  |  |  |  |
| Difference at 3 months | -0.01 | -0.05, 0.02 | 0.02 | -0.02, 0.05 | 0.03 | -0.01, 0.06 | 0.12 |
| Difference at 6 months | 0.01 | -0.03, 0.04 | 0.00 | -0.03, 0.04 | -0.01 | -0.04, 0.03 | 0.71 |
|  |  |  |  |  |  |  |  |
| **Waist circumference: Weight (cm/kg)** |  |  |  |  |  |  |  |
| Difference at 3 months | -0.04 | -0.27, 0.19 | 0.02 | -0.18, 0.23 | 0.06 | 0.00, 0.13 | 0.07 |
| Difference at 6 months | -0.01 | -0.26, 0.24 | 0.02 | -0.22, 0.26 | 0.03 | -0.12, 0.18 | 0.67 |
|  |  | |  | |  | |  |
|  | **Low Avocado** | | **High Avocado** | | **Between-group difference** | | **p-value** |
|  | **Mean** | **95% CI** | **Mean** | **95% CI** | **Mean** | **95% CI** |  |
| **Children only** |  |  |  |  |  |  |  |
| **Body mass index (kg/m^2^)** |  |  |  |  |  |  |  |
| Difference at 3 months | 0.25 | -1.71, 2.21 | 0.31 | -2.97, 3.59 | 0.06 | -0.47, 0.58 | 0.83 |
| Difference at 6 months | 0.53 | -1.58, 2.64 | 0.22 | -3.05, 3.49 | -0.31 | -1.18, 0.57 | 0.48 |
|  |  |  |  |  |  |  |  |
| **Waist circumference (cm)** |  |  |  |  |  |  |  |
| Difference at 3 months | 0.96 | -5.63, 7.55 | 3.37 | -5.06, 11.80 | 2.41 | -1.35, 6.17 | 0.20 |
| Difference at 6 months | 3.79 | -2.84, 10.42 | 4.00 | -4.24, 12.24 | 0.21 | -7.48, 7.90 | 0.96 |
|  |  |  |  |  |  |  |  |
| **Weight (kg)** |  |  |  |  |  |  |  |
| Difference at 3 months | 1.30 | -6.30, 8.91 | 1.28 | -8.58, 11.15 | -0.02 | -1.01, 0.97 | 0.97 |
| Difference at 6 months | 2.58 | -5.27, 10.43 | 3.05 | -6.91, 13.02 | 0.47 | -0.63, 1.58 | 0.40 |
|  |  |  |  |  |  |  |  |
| **Hip circumference (cm)** |  |  |  |  |  |  |  |
| Difference at 3 months | 2.62 | -3.20, 8.44 | 2.02 | -6.04, 10.07 | -0.60 | -2.34, 1.13 | 0.49 |
| Difference at 6 months | 4.63 | -1.53, 10.78 | 2.67 | -5.28, 10.61 | -1.96 | -9.74, 5.82 | 0.61 |
|  |  |  |  |  |  |  |  |
| **Waist circumference: Height (cm/cm)** |  |  |  |  |  |  |  |
| Difference at 3 months | 0.73 | 0.65, 0.80 | 0.81 | 0.72, 0.91 | 0.08 | 0.00, 0.16 | 0.04 |
| Difference at 6 months | 0.75 | 0.67, 0.82 | 0.82 | 0.73, 0.91 | 0.07 | -0.02, 0.16 | 0.13 |
|  |  |  |  |  |  |  |  |
| **Waist: Hip circumference (cm/cm)** |  |  |  |  |  |  |  |
| Difference at 3 months | -0.02 | -0.06, 0.02 | 0.02 | -0.04, 0.07 | 0.04 | -0.01, 0.08 | 0.14 |
| Difference at 6 months | 0.00 | -0.04, 0.03 | 0.02 | -0.03, 0.06 | 0.02 | -0.03, 0.07 | 0.43 |
|  |  |  |  |  |  |  |  |
| **Waist circumference: Weight (cm/kg)** |  |  |  |  |  |  |  |
| Difference at 3 months | -0.06 | -0.30, 0.18 | 0.02 | -0.23, 0.27 | 0.08 | -0.02, 0.18 | 0.10 |
| Difference at 6 months | -0.03 | -0.31, 0.25 | 0.04 | -0.29, 0.36 | 0.07 | -0.16, 0.30 | 0.57 |
|  |  |  |  |  |  |  |  |
|  |  |  |  |  |  |  |  |
|  | **Low Avocado** | | **High Avocado** | | **Between-group difference** | | **p-value** |
|  | **Mean** | **95% CI** | **Mean** | **95% CI** | **Mean** | **95% CI** |  |
| **Adolescents only** |  |  |  |  |  |  |  |
| **Body mass index (kg/m^2^)** |  |  |  |  |  |  |  |
| Difference at 3 months | 0.29 | -4.40, 5.00 | 0.08 | -3.24, 3.39 | -0.23 | -0.84, 0.39 | 0.46 |
| Difference at 6 months | -0.13 | -4.62, 4.37 | 0.90 | -2.55, 4.36 | 1.03 | -0.41, 2.48 | 0.15 |
|  |  |  |  |  |  |  |  |
| **Waist circumference (cm)** |  |  |  |  |  |  |  |
| Difference at 3 months | 1.00 | -12.17, 14.17 | 1.97 | -6.57, 10.51 | 0.97 | -3.28, 5.22 | 0.64 |
| Difference at 6 months | 2.60 | -10.71 | 0.32 | -7.76, 8.41 | -2.28 | -7.39, 2.83 | 0.36 |
|  |  |  |  |  |  |  |  |
| **Weight (kg)** |  |  |  |  |  |  |  |
| Difference at 3 months | 0.47 | -12.52, 13.45 | 0.05 | -9.04, 9.13 | -0.42 | -1.69, 0.84 | 0.50 |
| Difference at 6 months | 0.58 | -12.54, 13.71 | 2.61 | -6.86, 12.08 | 2.03 | -1.64, 5.70 | 0.27 |
|  |  |  |  |  |  |  |  |
| **Hip circumference (cm)** |  |  |  |  |  |  |  |
| Difference at 3 months | 1.41 | -8.04, 10.86 | 0.91 | -4.39, 6.21 | -0.50 | -3.69, 2.70 | 0.75 |
| Difference at 6 months | -0.45 | -10.23, 9.33 | 3.09 | -2.91, 9.09 | 3.54 | -1.19, 8.27 | 0.13 |
|  |  |  |  |  |  |  |  |
| **Waist circumference: Height (cm/cm)** |  |  |  |  |  |  |  |
| Difference at 3 months | 0.75 | 0.59, 0.91 | 0.80 | 0.69, 0.92 | 0.05 | -0.06, 0.17 | 0.33 |
| Difference at 6 months | 0.78 | 0.63, 0.92 | 0.78 | 0.67, 0.88 | 0.00 | -0.09, 0.08 | 0.99 |
|  |  |  |  |  |  |  |  |
| **Waist: Hip circumference (cm/cm)** |  |  |  |  |  |  |  |
| Difference at 3 months | 0.00 | -0.07, 0.07 | 0.01 | -0.05, 0.06 | 0.01 | -0.04, 0.05 | 0.69 |
| Difference at 6 months | 0.04 | -0.03, 0.11 | -0.02 | -0.07, 0.02 | -0.06 | -0.12, 0.00 | 0.04 |
|  |  |  |  |  |  |  |  |
| **Waist circumference: Weight (cm/kg)** |  |  |  |  |  |  |  |
| Difference at 3 months | 0.01 | -0.16, 0.18 | 0.03 | -0.04, 0.09 | 0.02 | -0.05, 0.09 | 0.58 |
| Difference at 6 months | 0.03 | -0.14, 0.20 | 0.00 | -0.08, 0.07 | -0.03 | -0.10, 0.04 | 0.35 |

^a^ The between-group difference is calculated by subtracting the value of the low avocado group from the high avocado group.

**Supplemental Table S2**. Changes in anthropometric variables, per protocol analysis adjusted for baseline body mass index, among the adolescents and children in the Effects of Avocado Intake on the Nutritional Status of Families Trial.

|  | **Within-group differences** | | **Within-group differences** | |  | |  |
| --- | --- | --- | --- | --- | --- | --- | --- |
|  | **Low Avocado** | | **High Avocado** | | **Between-group difference^a^** | | **p-value** |
|  | **Mean** | **95% CI** | **Mean** | **95% CI** | **Mean** | **95% CI** |  |
| **Children and Adolescents** |  |  |  |  |  |  |  |
| **Waist circumference (cm)** |  |  |  |  |  |  |  |
| Difference at 3 months | 0.97 | -1.54, 3.48 | 2.92 | -0.75, 6.59 | 1.95 | -1.04, 4.94 | 0.20 |
| Difference at 6 months | 3.31 | 0.03, 6.59 | 2.85 | -1.94, 7.63 | -0.46 | -5.92, 4.99 | 0.87 |
|  |  |  |  |  |  |  |  |
| **Weight (kg)** |  |  |  |  |  |  |  |
| Difference at 3 months | 1.02 | -2.71, 4.74 | 0.75 | -3.21, 4.70 | -0.27 | -1.11, 0.56 | 0.52 |
| Difference at 6 months | 1.91 | -1.76, 5.59 | 2.86 | -1.33, 7.05 | 0.95 | -0.91, 2.80 | 0.31 |
|  |  |  |  |  |  |  |  |
| **Hip circumference (cm)** |  |  |  |  |  |  |  |
| Difference at 3 months | 1.97 | -0.98, 4.92 | 1.65 | -1.23, 4.53 | -0.32 | -1.98, 1.34 | 0.70 |
| Difference at 6 months | 2.61 | -1.03, 6.25 | 3.04 | -1.59, 7.66 | 0.43 | -4.93, 5.78 | 0.87 |
|  |  |  |  |  |  |  |  |
| **Waist circumference: Height (cm/cm)** |  |  |  |  |  |  |  |
| Difference at 3 months | 0.74 | 0.69, 0.79 | 0.81 | 0.75, 0.86 | 0.07 | 0.03, 0.12 | <0.01 |
| Difference at 6 months | 0.75 | 0.71, 0.80 | 0.80 | 0.74, 0.86 | 0.05 | -0.02, 0.11 | 0.16 |
|  |  |  |  |  |  |  |  |
| **Waist: Hip circumference (cm/cm)** |  |  |  |  |  |  |  |
| Difference at 3 months | -0.01 | -0.04, 0.03 | 0.01 | -0.02, 0.05 | 0.02 | -0.01, 0.06 | 0.20 |
| Difference at 6 months | 0.01 | -0.02, 0.05 | 0.00 | -0.03, 0.04 | -0.01 | -0.05, 0.03 | 0.59 |
|  |  |  |  |  |  |  |  |
| **Waist circumference: Weight (cm/kg)** |  |  |  |  |  |  |  |
| Difference at 3 months | -0.04 | -0.21, 0.13 | 0.02 | -0.12, 0.17 | 0.06 | -0.01, 0.13 | 0.07 |
| Difference at 6 months | -0.01 | -0.20, 0.17 | 0.02 | -0.15, 0.19 | 0.03 | -0.11, 0.18 | 0.67 |
|  |  |  |  |  |  |  |  |
|  | **Low Avocado** | **High Avocado** | **Between-group difference** | **p-value** |  |  |  |
|  | **Mean** | **95% CI** | **Mean** | **95% CI** | **Mean** | **95% CI** |  |
| **Children only** |  |  |  |  |  |  |  |
| **Waist circumference (cm)** |  |  |  |  |  |  |  |
| Difference at 3 months | 0.96 | -2.20, 4.11 | 3.54 | -1.93, 9.01 | 2.58 | -1.18, 6.34 | 0.17 |
| Difference at 6 months | 3.64 | -0.88, 8.15 | 4.50 | -2.73, 11.73 | 0.86 | -7.38, 9.10 | 0.83 |
|  |  |  |  |  |  |  |  |
| **Weight (kg)** |  |  |  |  |  |  |  |
| Difference at 3 months | 1.30 | -2.03, 4.64 | 1.28 | -2.74, 5.31 | -0.02 | -0.96, 0.92 | 0.97 |
| Difference at 6 months | 2.58 | -0.83, 5.99 | 3.05 | -0.97, 7.08 | 0.47 | -0.50, 1.44 | 0.33 |
|  |  |  |  |  |  |  |  |
| **Hip circumference (cm)** |  |  |  |  |  |  |  |
| Difference at 3 months | 2.24 | -0.44, 4.92 | 2.14 | -0.65, 4.92 | -0.10 | -1.84, 1.63 | 0.90 |
| Difference at 6 months | 4.00 | -0.44, 8.44 | 3.00 | -3.39, 9.39 | -1.00 | -8.68, 6.68 | 0.79 |
|  |  |  |  |  |  |  |  |
| **Waist circumference: Height (cm/cm)** |  |  |  |  |  |  |  |
| Difference at 3 months | 0.73 | 0.67, 0.78 | 0.81 | 0.74, 0.88 | 0.08 | 0.03, 0.14 | <0.01 |
| Difference at 6 months | 0.75 | 0.69, 0.80 | 0.82 | 0.74, 0.90 | 0.07 | -0.02, 0.16 | 0.11 |
|  |  |  |  |  |  |  |  |
| **Waist: Hip circumference (cm/cm)** |  |  |  |  |  |  |  |
| Difference at 3 months | -0.01 | -0.05, 0.02 | 0.02 | -0.04, 0.07 | 0.03 | -0.02, 0.08 | 0.21 |
| Difference at 6 months | 0.00 | -0.03, 0.04 | 0.02 | -0.03, 0.07 | 0.02 | -0.04, 0.07 | 0.53 |
|  |  |  |  |  |  |  |  |
| **Waist circumference: Weight (cm/kg)** |  |  |  |  |  |  |  |
| Difference at 3 months | -0.06 | -0.24, 0.12 | 0.02 | -0.14, 0.18 | 0.08 | -0.02, 0.18 | 0.10 |
| Difference at 6 months | -0.03 | -0.24, 0.18 | 0.04 | -0.19, 0.26 | 0.07 | -0.17, 0.30 | 0.57 |
|  |  |  |  |  |  |  |  |
|  | **Low Avocado** | **High Avocado** | **Between-group difference** | **p-value** |  |  |  |
|  | **Mean** | **95% CI** | **Mean** | **95% CI** | **Mean** | **95% CI** |  |
| **Adolescents only** |  |  |  |  |  |  |  |
| **Waist circumference (cm)** |  |  |  |  |  |  |  |
| Difference at 3 months | 1.00 | -2.96, 4.96 | 1.97 | -1.93, 5.87 | 0.97 | -3.88, 5.82 | 0.68 |
| Difference at 6 months | 2.60 | -1.58, 6.78 | 0.32 | -3.99, 4.64 | -2.28 | -7.15, 2.59 | 0.34 |
|  |  |  |  |  |  |  |  |
| **Weight (kg)** |  |  |  |  |  |  |  |
| Difference at 3 months | 0.47 | -4.18, 5.11 | 0.05 | -3.42, 3.51 | -0.42 | -1.90, 1.05 | 0.56 |
| Difference at 6 months | 0.58 | -4.43, 5.59 | 2.61 | -1.93, 7.15 | 2.03 | -2.69, 6.75 | 0.39 |
|  |  |  |  |  |  |  |  |
| **Hip circumference (cm)** |  |  |  |  |  |  |  |
| Difference at 3 months | 1.41 | -1.37, 4.19 | 0.91 | -1.86, 3.68 | -0.50 | -4.13, 3.14 | 0.78 |
| Difference at 6 months | -0.45 | -3.99, 3.09 | 3.09 | 0.00, 6.17 | 3.54 | -1.40, 8.48 | 0.15 |
|  |  |  |  |  |  |  |  |
| **Waist circumference: Height (cm/cm)** |  |  |  |  |  |  |  |
| Difference at 3 months | 0.75 | 0.66, 0.84 | 0.80 | 0.74, 0.87 | 0.05 | -0.01, 0.12 | 0.11 |
| Difference at 6 months | 0.78 | 0.70, 0.85 | 0.78 | 0.72, 0.84 | 0.00 | -0.07, 0.07 | 0.99 |
|  |  |  |  |  |  |  |  |
| **Waist: Hip circumference (cm/cm)** |  |  |  |  |  |  |  |
| Difference at 3 months | 0.00 | -0.04, 0.05 | 0.01 | -0.03, 0.05 | 0.01 | -0.04, 0.06 | 0.73 |
| Difference at 6 months | 0.04 | -0.02, 0.09 | -0.02 | -0.06, 0.02 | -0.06 | -0.11, -0.02 | 0.02 |
|  |  |  |  |  |  |  |  |
| **Waist circumference: Weight (cm/kg)** |  |  |  |  |  |  |  |
| Difference at 3 months | 0.01 | -0.14, 0.16 | 0.03 | -0.03, 0.09 | 0.02 | -0.05, 0.09 | 0.58 |
| Difference at 6 months | 0.03 | -0.12, 0.17 | 0.00 | -0.07, 0.06 | -0.03 | -0.10, 0.03 | 0.30 |

^a^ The between-group difference is calculated by subtracting the value of the low avocado group from the high avocado group.

**Supplemental Table S3**. Changes in anthropometric variables, per protocol analysis adjusted for baseline calorie intake and adherence, among the adolescents and children in the Effects of Avocado Intake on the Nutritional Status of Families Trial.

|  | **Within-group differences** | | **Within-group differences** | |  | |  |
| --- | --- | --- | --- | --- | --- | --- | --- |
|  | **Low Avocado** | | **High Avocado** | | **Between-group difference^a^** | | **p-value** |
|  | **Mean** | **95% CI** | **Mean** | **95% CI** | **Mean** | **95% CI** |  |
| **Children and Adolescents** |  |  |  |  |  |  |  |
| **Body mass index (kg/m^2^)** |  |  |  |  |  |  |  |
| Difference at 3 months | -0.27 | -2.47, 1.93 | -0.21 | -2.40, 1.99 | 0.06 | -0.36, 0.49 | 0.76 |
| Difference at 6 months | -0.31 | -2.48, 1.86 | -0.52 | -2.94, 1.90 | -0.21 | -1.09, 0.67 | 0.64 |
|  |  |  |  |  |  |  |  |
| **Waist circumference (cm)** |  |  |  |  |  |  |  |
| Difference at 3 months | -0.91 | -7.60, 5.78 | -2.92 | -9.10, 3.26 | -2.01 | -5.12, 1.10 | 0.20 |
| Difference at 6 months | -3.31 | -9.97, 3.35 | -2.85 | -9.26, 3.56 | 0.46 | -5.15, 6.08 | 0.87 |
|  |  |  |  |  |  |  |  |
| **Weight (kg)** |  |  |  |  |  |  |  |
| Difference at 3 months | -1.03 | -9.82, 7.76 | -0.75 | -8.17, 6.68 | 0.28 | -0.56, 1.13 | 0.51 |
| Difference at 6 months | -1.92 | -10.71, 6.88 | -2.86 | -11.32, 5.60 | -0.95 | -2.79, 0.90 | 0.31 |
|  |  |  |  |  |  |  |  |
| **Hip circumference (cm)** |  |  |  |  |  |  |  |
| Difference at 3 months | -2.02 | -8.90, 4.87 | -1.64 | -7.65, 4.35 | 0.36 | -1.28, 2.01 | 0.66 |
| Difference at 6 months | -2.61 | -9.44, 4.22 | -3.03 | 9.68, 3.61 | -0.43 | -6.10, 5.25 | 0.88 |
|  |  |  |  |  |  |  |  |
| **Waist circumference: Height (cm/cm)** |  |  |  |  |  |  |  |
| Difference at 3 months | -0.74 | -0.80, -0.67 | -0.81 | -0.88, -0.74 | -0.07 | -0.13, 0.00 | 0.04 |
| Difference at 6 months | -0.76 | -0.82, -0.70 | -0.80 | -0.87, -0.74 | -0.05 | -0.11, 0.02 | 0.19 |
|  |  |  |  |  |  |  |  |
| **Waist: Hip circumference (cm/cm)** |  |  |  |  |  |  |  |
| Difference at 3 months | 0.01 | -0.02, 0.05 | -0.01 | -0.05, 0.02 | -0.02 | -0.06, 0.12 | 0.19 |
| Difference at 6 months | -0.01 | -0.05, 0.02 | 0.00 | -0.04, 0.03 | 0.01 | -0.03, 0.05 | 0.59 |
|  |  |  |  |  |  |  |  |
| **Waist circumference: Weight (cm/kg)** |  |  |  |  |  |  |  |
| Difference at 3 months | 0.04 | -0.19, 0.28 | -0.02 | -0.21, 0.17 | -0.07 | -0.14, 0.01 | 0.07 |
| Difference at 6 months | 0.01 | -0.24, 0.26 | -0.02 | -0.26, 0.22 | -0.03 | -0.18, 0.12 | 0.68 |
|  |  |  |  |  |  |  |  |
|  | **Low Avocado** | **High Avocado** | **Between-group difference** | **p-value** | **Between-group difference^a^** | |  |
|  | **Mean** | **95% CI** | **Mean** | **95% CI** | **Mean** | **95% CI** | **p-value** |
| **Children only** |  |  |  |  |  |  |  |
| **Body mass index (kg/m^2^)** |  |  |  |  |  |  |  |
| Difference at 3 months | -0.25 | -2.08, 1.58 | -0.31 | -3.34, 2.73 | -0.06 | -0.60, 0.48 | 0.84 |
| Difference at 6 months | -0.53 | -2.56, 1.51 | -0.22 | -3.03, 2.59 | 0.31 | -0.61, 1.23 | 0.50 |
|  |  |  |  |  |  |  |  |
| **Waist circumference (cm)** |  |  |  |  |  |  |  |
| Difference at 3 months | -0.96 | -6.85, 4.93 | -3.54 | -11.51, 4.43 | -2.58 | -6.62, 1.45 | 0.20 |
| Difference at 6 months | -6.64 | -10.23, 2.96 | -4.50 | -12.12, 3.12 | -0.86 | -9.18, 7.45 | 0.84 |
|  |  |  |  |  |  |  |  |
| **Weight (kg)** |  |  |  |  |  |  |  |
| Difference at 3 months | -1.30 | -8.09, 5.49 | -1.28 | -10.08, 7.52 | 0.02 | -0.98, 1.02 | 0.97 |
| Difference at 6 months | -2.58 | -9.88, 4.71 | -3.05 | -11.76, 5.65 | -0.47 | -1.61, 0.66 | 0.41 |
|  |  |  |  |  |  |  |  |
| **Hip circumference (cm)** |  |  |  |  |  |  |  |
| Difference at 3 months | -2.24 | -7.68, 3.20 | -2.14 | -9.50, 5.23 | 0.10 | -1.55, 1.76 | 0.90 |
| Difference at 6 months | -4.00 | -10.24, 2.24 | -3.00 | -10.28, 4.28 | 1.00 | -7.56, 9.56 | 0.81 |
|  |  |  |  |  |  |  |  |
| **Waist circumference: Height (cm/cm)** |  |  |  |  |  |  |  |
| Difference at 3 months | -0.73 | -0.80, -0.66 | -0.81 | -0.90, -0.72 | -0.08 | -0.16, 0.00 | 0.05 |
| Difference at 6 months | -0.75 | -0.82, -0.68 | -0.82 | -0.90, -0.73 | -0.07 | -0.17, 0.02 | 0.14 |
|  |  |  |  |  |  |  |  |
| **Waist: Hip circumference (cm/cm)** |  |  |  |  |  |  |  |
| Difference at 3 months | 0.01 | -0.02, 0.05 | -0.02 | -0.07, 0.04 | -0.03 | -0.08, 0.02 | 0.21 |
| Difference at 6 months | 0.00 | -0.04, 0.04 | -0.02 | -0.07, 0.03 | -0.02 | -0.07, 0.04 | 0.54 |
|  |  |  |  |  |  |  |  |
| **Waist circumference: Weight (cm/kg)** |  |  |  |  |  |  |  |
| Difference at 3 months | 0.06 | -0.17, 0.29 | -0.02 | -0.26, 0.22 | -0.08 | -0.19, 0.02 | 0.11 |
| Difference at 6 months | 0.03 | -0.24, 0.30 | -0.04 | -0.35, 0.28 | -0.07 | -0.30, 0.17 | 0.58 |
|  |  |  |  |  |  |  |  |
|  | **Low Avocado** | **High Avocado** | **Between-group difference** | **p-value** | **Between-group difference^a^** | |  |
|  | **Mean** | **95% CI** | **Mean** | **95% CI** | **Mean** | **95% CI** | **p-value** |
| **Adolescents only** |  |  |  |  |  |  |  |
| **Body mass index (kg/m^2^)** |  |  |  |  |  |  |  |
| Difference at 3 months | -0.31 | -3.66, 3.05 | -0.08 | -3.18, 3.03 | 0.23 | -0.50, 0.96 | 0.52 |
| Difference at 6 months | 0.13 | -4.23, 4.49 | -0.90 | -4.35, 2.54 | -1.03 | -2.91, 0.84 | 0.27 |
|  |  |  |  |  |  |  |  |
| **Waist circumference (cm)** |  |  |  |  |  |  |  |
| Difference at 3 months | -0.80 | -12.13, 10.53 | -1.97 | -10.33, 6.39 | -1.17 | -6.05, 3.71 | 0.62 |
| Difference at 6 months | -2.60 | -15.47, 10.27 | -0.32 | -8.56, 7.91 | 2.28 | -3.11, 7.66 | 0.39 |
|  |  |  |  |  |  |  |  |
| **Weight (kg)** |  |  |  |  |  |  |  |
| Difference at 3 months | -0.45 | -11.23, 10.32 | -0.04 | -7.84, 7.75 | 0.41 | -1.08, 1.90 | 0.58 |
| Difference at 6 months | -0.58 | -13.90, 12.73 | -2.61 | -12.02, 6.80 | -2.02 | -6.79, 2.74 | 0.39 |
|  |  |  |  |  |  |  |  |
| **Hip circumference (cm)** |  |  |  |  |  |  |  |
| Difference at 3 months | -1.50 | -8.27, 5.27 | -0.91 | -6.08, 4.25 | 0.59 | -3.32, 4.50 | 0.76 |
| Difference at 6 months | 0.45 | -9.20, 10.10 | -3.09 | -9.20, 3.02 | -3.54 | -8.59, 1.52 | 0.16 |
|  |  |  |  |  |  |  |  |
| **Waist circumference: Height (cm/cm)** |  |  |  |  |  |  |  |
| Difference at 3 months | -0.76 | -0.91, -0.62 | -0.80 | -0.92, -0.69 | -0.04 | -0.16, 0.08 | 0.49 |
| Difference at 6 months | -0.78 | -0.91, -0.64 | -0.78 | -0.88, 0.78 | 0.00 | -0.10, 0.10 | 0.99 |
|  |  |  |  |  |  |  |  |
| **Waist: Hip circumference (cm/cm)** |  |  |  |  |  |  |  |
| Difference at 3 months | 0.00 | -0.07, 0.07 | -0.01 | -0.07, 0.05 | -0.01 | -0.06, 0.04 | 0.64 |
| Difference at 6 months | 0.04 | -0.03, 0.11 | -0.02 | -0.07, 0.02 | -0.06 | -0.11, -0.01 | 0.03 |
|  |  |  |  |  |  |  |  |
| **Waist circumference: Weight (cm/kg)** |  |  |  |  |  |  |  |
| Difference at 3 months | -0.01 | -0.18, 0.16 | -0.03 | -0.09, 0.03 | -0.02 | -0.09, 0.05 | 0.51 |
| Difference at 6 months | -0.03 | -0.21, 0.15 | 0.00 | -0.07, 0.08 | 0.03 | -0.04, 0.10 | 0.34 |

^a^ The between-group difference is calculated by subtracting the value of the low avocado group from the high avocado group.
